# Mitochondrial Genome Comparison and Phylogenetic Analysis of Four Species of Dung Beetles (Coleoptera: Scarabaeidae: Scarabaeinae)

**DOI:** 10.1002/ece3.71906

**Published:** 2025-08-05

**Authors:** Honghui Zhang, Qiuju He, Zhiyong Zhao, Bin Zhang, Jun Zhou, Chengye Wang, Chuanhui Yi, Min Zhao

**Affiliations:** ^1^ Institute of Highland Forest Science Chinese Academy of Forestry Kunming Yunnan China; ^2^ College of Forestry, Southwest Forestry University Kunming Yunnan China; ^3^ Yunnan Animal Science and Veterinary Institute Kunming Yunnan China; ^4^ Key Laboratory of Breeding and Utilization of Resource Insects, National Forestry and Grassland Administration Kunming Yunnan China; ^5^ Yunnan Academy of Biodiversity, Southwest Forestry University Kunming Yunnan China; ^6^ Yunnan Key Laboratory of Breeding and Utilization of Resource Insects Kunming China

**Keywords:** dung beetle, mitochondrial genome, phylogeny, Scarabaeinae

## Abstract

Dung beetles, despite their well‐documented ecological significance, remain surprisingly underrepresented in molecular biology research, with a notable paucity of genomic and molecular data available for this ecologically crucial insect group. We sequenced and analyzed the whole mitochondrial genomes of four species of dung beetles, including *Catharsius molossus*, *Liatongus bucerus*, *Copris magicus*, and *Onitis falcatus* from the Scarabaeinae subfamily. The results showed that the mitochondrial genome sizes of the four dung beetle species ranged from 14,977 bp to 18,425 bp. Among them, the mitochondrial genome structure, base composition, codon usage, and gene arrangement of *L. bucerus* and *C. magicus* were relatively conserved, consistent with other Coleoptera species. However, *C. magicus* exhibited a unique rearrangement, with the *trnT* and *trnP* genes undergoing positional swapping, resulting in the formation of a *trnP‐trnT* tRNA gene block. In *L. bucerus* , a long‐distance translocation of the *trnI* gene and rearrangement between the *trnS2* and *nad1* genes occurred, leading to the formation of a *trnS2‐trnI* tRNA gene block. This represents the first identification of such rearrangements in Scarabaeidae. The phylogenetic reconstruction of mitochondrial protein‐coding genes in 63 species of Scarabaeinae revealed significant insights into their evolutionary relationships. It supported multilineage for the tribes Coprini and Onthophagini, and monophyly for the tribes Onitini and Oniticellini. Coprini maintained close affinities with Deltochilini, Dichotomiini, Phanaeini, Ateuchini, and Eurysternini, with Phanaeini as a sister group to each other, and the phylogenetic relationships among the tribes supported the existing findings. It clarified the position of four dung beetles in their respective clades and genera. This study could enrich the genetic data of Scarabaeidae and establishes a foundation for further understanding of the evolutionary relationships of dung beetles.

## Graphical abstract

Dung beetles have important ecological and economical values; however, the molecular research of the taxonomic group is very limited. We sequenced mitochondrial genomes of four Scarabaeinae species, with sizes from 14,977 to 18,425 bp, and identified novel gene rearrangements. Phylogenetic analysis of 63 Scarabaeinae species supported multilineage origins for Coprini and Onthophagini tribes, monophyly for Onitini and Oniticellini, and clarified evolutionary ties between tribes, aligning with existing classifications. This work enriched Scarabaeidae genetic data and advanced understanding of dung beetle evolution, and provided a foundation for future molecular and ecological studies.

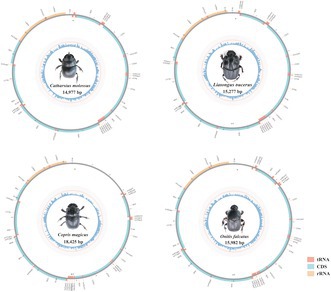

## 1 Introduction

Dung beetles belong to the subfamilies Scarabaeinae and Aphodiinae within the family Scarabaeidae of the order Coleoptera. With approximately 7000 species, they form a diverse group of insects (Daniel and Davis 2024) and are widespread on all continents except Antarctica (Bai and Yang 2010; Tarasov and Génier 2015). These insects are primarily dung‐feeding or scavenging, playing a crucial role as decomposers in the Earth's ecosystem. However, in recent years, habitat destruction and the significant decline of grazing lands have posed serious threats to their survival; urgent conservation efforts are needed.

The taxonomic study of Scarabaeinae has been particularly intriguing because of its long history of controversy. In 1949, Janssens (1949) divided the Scarabaeinae into six tribes based on morphology; they are the tribes Coprini, Eurysternini, Oniticellini, Onitini, Onthophagini, and Scarabaeini. Till 1963, Balthasar (1963) proposed an important dichotomous framework for the division of the dung beetle taxon into two major groups: the Coprinae and the Scarabaeinae. Halffter and Martínez (1966, 1967, 1968, 1977) attempted to introduce internal genitalia characters for species delimitation, but the method did not become mainstream due to the lack of widespread acceptance and sufficient evidence to support it. As studies progressed, Hanski and Cambefort (1991) supported Balthasar's dichotomy based on the natural or monophyletic taxa of the organisms, defining the Coprinae as a group of tunnel‐digging beetles and the Scarabaeinae as a group of dung‐ball‐rolling beetles. However, this classification lacks phylogenetic evidence to support it and is considered an intuitive one. Luzzatto (1994) confirmed the dichotomy between Coprinae and Scarabaeinae to some extent by comparing male and female genital structures in 1994. Yet, the presence of Phanaeini and Onitini features in Eucraniini was found, and the classification of Deltochilini and Ateuchini was questioned. Montreuil (1998) studied the tribes Ateuchini and Coprini using 42 adult morphometric characters and found that both were non‐monophyletic, suggesting that the subfamily classification requires major revision. Subsequent studies further revealed that Dichotomiini and Canthonini in Balthasar's classification were also non‐monophyletic (Philips et al. 2004), which strongly motivated the need for a revision of the taxonomic system. Early taxonomic research on dung beetles mainly relied on morphology (including genital structure), behavior, and biology, and each change in classification criteria caused significant changes and new conflicts in the classification system.

Molecular biology methods brought new perspectives to dung beetle taxonomy, but also posed additional challenges to Balthasar's classical framework; for instance, phylogenetic analyses based on mitochondrial genes (*COI* and *COII*) and nuclear gene sequences showed inconsistencies in sisterhood relationships among tribes other than the Sisyphini and Gymnopleurini tribes (Villalba et al. 2002; Ocampo and Hawks 2006). Tribes, serving as an intermediate rank between subfamily and genus, play a minor yet crucial role in the detailed classification of certain large families or subfamilies. Daniel and Davis (2024) conducted a comprehensive review of the tribe classification of Scarabaeinae based on molecular markers, morphological traits, biogeographical distribution, and paleontological evidence. A compilation of the currently recognized classification system of dung beetles, which includes 20 tribes, as well as some new groups, such as the tribes Ateuchini, Byrrhidiini, Endroedyolini, Epactoidini, Odontolomini, and Parachoriini. Jeong et al. (2020) reconstructed a phylogenetic tree using 13 mitochondrial protein‐coding genes (PCGs), and the results supported the non‐monophyly of the tribe Onthophagini. Mello et al. (2021) reconstructed a phylogenetic tree using mitochondrial genome data obtained by second‐generation sequencing by Bayesian inference (BI), and the results showed that the tribe Phanaeini was a monophyletic group and supported the hypothesis that Coprini is its sister group. A phylogenetic tree constructed using the mitochondrial genome supports the polyphyletic nature of the Onthophagini and Ateuchini tribes by Guo et al. (2022). The reorganized classification system proposed by Daniel and Davis (2024) provided support for a global phylogenetic classification for Scarabaeinae species. This system offered varying levels of statistical support for the delimitation of eight tribes within Scarabaeinae, as proposed by Tarasov and Dimitrov (2016). Nevertheless, establishing phylogenetically consistent tribal definitions remains a significant challenge.

Mitochondrial genome, characterized by its conserved arrangement, small molecular weight, ease of isolation and extraction, and matrilineal inheritance, has facilitated diverse applications in taxonomic identification of related species, molecular phylogenetics, population evolution, and genetic and phylogenetic reconstruction (Nie et al. 2018; Wang et al. 2013; Wei and Chen 2011; Cameron 2014). The mitochondrial genome offers a larger molecular dataset than a single gene, providing greater robustness in phylogenetic analysis (Nie et al. 2018), which has been widely used for reconstructing phylogenetic trees of Scarabaeidae in recent years (Hu et al. 2023; Ayivi et al. 2021; Yu et al. 2023). Conducting phylogenetic analysis based on mitochondrial genome data, Hu et al. (2023) indicated that the subfamilies of Scarabaeinae, Cetoniinae, Rutelinae, Dynastinae, and Melolonthinae are monophyletic groups. Ayivi et al. (2021) analyzed 18 new phytophagous Scarabaeidae mitochondrial genomes, recovered the monophyly of Rutelinae, Cetoniinae, Dynastinae, and Sericinae, and the non‐monophyly of Melolonthinae. However, there are so many insects in the family Scarabaeidae, and many genera not only lack reports of complete mitochondrial genomes, but the evolutionary relationships among these genera are also unclear. Yu et al. (2023) present the first mitochondrial genome of a long‐armed scarab beetle *Euchirus longimanus*, from the type genus *Euchirus*, reconfirm the phylogenetic position of Euchirini within the paraphyletic Melolonthinae, and elucidate the phylogenetic relationship among all three genera: Propomacrus + (Cheirotonus + Euchirus). Refining phylogenetic relationships through extensive mitochondrial genomic data is especially valuable (Jeong et al. 2020; Mello et al. 2021).

In this study, we sequenced the whole mitochondrial genomes of four dung beetle species— *Catharsius molossus* , *Liatongus bucerus* , *C. magicus*, and *Onitis falcatus* —collected in Yunnan Province, China. Among them, the mitochondrial genomes of *L. bucerus* and *C. magicus* were sequenced for the first time, and different types of gene rearrangements were found in these two dung beetles. The uniqueness of gene rearrangements in a given evolutionary clade can serve as “synapomorphies”, which is clearly a potential phylogenetic marker that is crucial for the phylogenetic classification of species. In addition, we analyzed the structural characteristics of four species of dung beetles and constructed a phylogenetic tree based on the mitochondrial genome sequences of 63 Scarabaeinae species, using three Aphodiinae species as outgroups, mitochondrial whole genome data for 59 Scarabaeinae species were obtained from NCBI, covering the tribes Ateuchini, Coprini, Deltochilini, Dichotomiini, Eurysternini, Oniticellini, Onitini, Onthophagini, and Phanaeini, which are nine tribes (45% of the 20 recognized tribes (Daniel and Davis 2024)) with relatively stable taxonomic relationships (Tarasov and Dimitrov 2016). However, there are 11 tribes (Byrrhidiini, Endroedyolini, Epactoidini, Epilissini, Epirinini, Eucraniini, Gymnopleurini, Odontolomini, Parachoriini, Scarabaeini and Sisyphini) still lacking complete mitochondrial genome data. We added the sequencing of *C. molossus* and *C. magicus* from the tribe Coprini, *L. bucerus* from the tribe Oniticellini, and *O. falcatus* from the tribe Onitini, which provide newly representative genera within the three tribes. This study aims to deepen the understanding of the dung beetle mitochondrial genome by evaluating the reliability and support of PCGs in constructing phylogenetic relationships among Scarabaeinae tribes. It seeks to provide data to support dung beetle species conservation and establish a foundation for further insights into their taxonomic and evolutionary relationships.

## 2 Materials and Methods

### 2.1 Source of Test Material

Adult dung beetles were collected between July and August 2023 from Yunnan Province, China, by digging the cave of dung beetles in the wild to obtain live individuals. Specifically, *L. bucerus* and *C. magicus* were collected from Xundian District, Kunming City. *O. falcatus* was collected from Linxiang District, Lincang City, and *C. molossus* was collected from Tengchong City. Samples were treated with 75% ethanol three times, followed by transferring to anhydrous ethanol. They were then stored in an ultra‐low temperature refrigerator at −80°C for preservation.

### 2.2 Ethics Statement

Adult dung beetle material was used in this study. The collecting of dung beetle specimens did not require a special license. Dung beetle specimens were deposited in the herbarium of the Institute of Highland Forest Science, Chinese Academy of Forestry, Kunming, China. This study was conducted following all relevant institutional, national, and international guidelines and policies.

### 2.3 DNA Extraction and Sequencing

The total DNA from the thoracic muscle tissue of adult dung beetles was extracted using the Cetyltrimethylammonium bromide (CTAB) method. Subsequently, a sequencing library was constructed using Sequencing By Synthesis (SBS) technology and the Illumina HiSeq high‐throughput sequencing platform. Beijing Tsingke Biotechnology Co. Ltd. performed the sequencing of qualified DNA samples.

### 2.4 Mitogenome Assembly, Annotation, and Characteristics Analysis

The mitochondrial genome sequences were assembled using a two‐step strategy to ensure accuracy and completeness. First, de novo assembly was performed using SPAdes v3.11.1 (Bankevich et al. 2012), a widely used general‐purpose genome assembler that can recover organelle sequences from total genomic data. However, SPAdes is not specifically optimized for circular organelle genomes and may yield fragmented or contaminated assemblies. Therefore, we further applied GetOrganelle (https://github.com/Kinggerm/GetOrganelle), which is tailored for accurate assembly of circular organelle genomes, to extract, assemble, and verify the mitochondrial genomes with high precision. This tool uses a graph‐based approach and reads filtration steps to reconstruct complete, circularized organellar genomes. The combined use of both assemblers allowed us to cross‐validate results and ensure high assembly quality. The SPAdes contigs were also extended using SSPACE v2.1.1, and circularization of the final assembly was confirmed through alignment and manual verification. To ensure accuracy, the assembled genome sequence underwent evaluation for circularization. The circularized genome sequence was aligned with the NCBI species classification database and underwent manual correction as needed. The tRNA genes and their secondary structures were identified and predicted using tRNAscan‐SE software (Laslett and Canbäck 2008). Mitochondrial gene prediction was performed using the online annotation tool Galaxy (Bernt et al. 2013). MEGA v11 (Tamura et al. 2021) was utilized to calculate the basic sequence composition, base content, initiation and termination codons, as well as their respective positions within the PCGs. Base deviations were calculated using the formulas AT‐skew = (A–T)/(A + T) and GC‐skew = (G–C)/(G + C) (Perna and Kocher 1995). Codon numbers and relative synonymous codon usage information for PCGs were calculated using PhyloSuite v1.2.3 software (Zhang et al. 2020). The 13 PCGs sequences from the four sequenced species were pairwise compared using DnaSP v6.0 (Rozas et al. 2017). This analysis computed the synonymous substitution rate (Ks) and non‐synonymous substitution rate (Ka). Additionally, the Ka/Ks ratios were calculated to assess the evolutionary pressures acting on these genes. When Ka/Ks > 1, the gene is subjected to positive selection; when Ka/Ks = 1, the gene is subjected to neutral selection; when Ka/Ks < 1, the gene is subjected to purifying selection, and when the Ka/Ks value is closer to 1, the selection pressure on the gene is smaller (Zhang et al. 2006). Tandem repeat sequences in the AT‐rich region were identified using the Tandem Repeats Finder tool (http://tandem.bu.edu/trf/trf.html). The final visualization and mapping of the circular mitochondrial genome of the tested dung beetle were performed using OGDRAW v1.3.1 (Greiner et al. 2019).

### 2.5 Taxonomic Sampling

In order to verify the phylogenetic relationships among tribes and genus within Scarabaeinae, this study screened the mitochondrial genomes of the species from the NCBI database (https://www.ncbi.nlm.nih.gov) for the construction of the phylogenetic tree, and the need for the selection of species in the inner and outer groups fulfilled the following conditions: (i) the mitochondrial genomes of the ingroup and outgroup species need to contain a complete set of 37 gene fragments with complete and correct annotation information; (ii) the ingroup species should be selected from those currently recognized as belonging to the taxonomic system of Scarabaeinae, with reference to the taxonomic system of dung beetles compiled by Daniel and Davis (2024); (iii) the outgroups were selected from the species belonging to Aphodiinae, which are closely related to Scarabaeinae. Based on the study by Guo et al. (2022), three species of Scarabaeidae that have not been identified to species names meet the selection criterion. Among them, Scarabaeidae sp. BMNH 1274752 belongs to the Deltochilini tribe with a bootstrap value of 65 and a posterior probability of 0.93, Scarabaeidae sp. BMNH 1274750 and Scarabaeidae sp. BMNH 1274753 belong to the Ateuchini tribe (with bootstrap values of 100 and posterior probability of 1, respectively). Based on the above conditions, a total of 59 mitochondrial genome information of endogamous species and 3 mitochondrial genome information of exogamous species were screened from the NCBI database.

### 2.6 Phylogenetic Analyses

The phylogenetic trees were constructed using the nucleotide sequences of the 13 PCGs from the mitochondrial genomes. Reference sequences included whole mitochondrial genomes from 59 representative species of Scarabaeinae and four newly sequenced dung beetle species (refer to Table 1). The maximum likelihood (ML) and BI methods were employed for tree construction, with *Aphodius elegans*, *Aphodius foetens*, and *Oxyomus sylvestris* from Aphodiinae serving as outgroups. Multiple sequence alignment of the PCGs was conducted using MAFFT v7.526 (Katoh and Standley 2013). The alignment results were refined and screened using Gblocks v0.91b (Talavera and Castresana 2007) to remove poorly aligned positions and regions of uncertain homology. Subsequently, the aligned and screened gene sequences were concatenated using SequenceMatrix v1.7.8 (Vaidya et al. 2011), resulting in a dataset comprising the concatenated sequences of the 13 PCGs. PartitionFinder2 and ModelFinder plugins within PhyloSuite v1.2.3 (Lanfear et al. 2017) were employed to optimize the tree‐building dataset, determining the best alternative model, specifically the JC + I + G model, for phylogenetic analysis. Based on the phylogenetic tree generated by PhyloSuite software, the ML phylogenetic tree was constructed using IQ‐TREE v1.6.8 (Lam‐Tung et al. 2015). The analysis included 1000 bootstrap (BS) replicates to assess node support. Additionally, the BI phylogenetic tree was constructed using MrBayes v3.2.7 (Ronquist et al. 2012). The analysis was set up with four independent Markov chains running simultaneously for 2 million generations. Sampling occurred every 100 generations, with the first 25% of samples discarded as burn‐in. Convergence was assessed by ensuring that the potential scale reduction factor (PSRF) for all parameters was close to 1.0, indicating reliable results. A consensus tree was constructed from the remaining samples, and Bayesian posterior probability (PP) values were calculated for each node to indicate confidence in the tree topology. The final phylogenetic tree was landscaped using FigTree v.1.4.3 (http://tree.bio.ed.ac.uk/software/figtree).

**TABLE 1:** Information on the mitochondrial genomes used to construct phylogenetic tree.

Family	Subfamily	Species	GenBank number	Length (bp)	References
Scarabaeidae	Aphodiinae	*Aphodius elegans*	PQ083081.1	16,447	Unpublished
		*Aphodius foetens*	KX087240.1	15,907	Guo et al. (2022)
		*Oxyomus sylvestris*	KX087329.1	12,839	Guo et al. (2022)
Scarabaeidae	Scarabaeinae	*Bubas bison*	KU739470.1	13,466	Gunter et al. (2016)
		*Bubas bubalus*	KU739469.1	16,035	Gunter et al. (2016)
		*Caccobius nigritulus*	KU739484.1	15,039	Gunter et al. (2016)
		*Canthidium* sp. DPP‐2018	MG253260.1	15,517	Guo et al. (2022)
		*Catharsius molossus*	MT548776.1	15,036	Unpublished
		*Catharsius molossus*	PQ179711	14,977	This study
		*Cheironitis hoplosternus*	KU739450.1	14,924	Gunter et al. (2016)
		*Copris magicus*	PQ179712	18,425	This study
		*Copris tripartitus*	NC_045923.1	15,457	Jeong et al. (2020)
		*Coprophanaeus* sp. BMNH679884	KU739465.1	15,554	Gunter et al. (2016)
		*Dichotomius schiffleri*	NC_039689.1	14,802	Amorim et al. (2017)
		*Digitonthophagus gazella*	KU739497.1	15,302	Gunter et al. (2016)
		*Drepanocerus kirbyi*	KU739491.1	15,780	Gunter et al. (2016)
		*Euoniticellus fulvus*	KU739453.1	15,494	Gunter et al. (2016)
		*Euoniticellus intermedius*	KU739490.1	15,578	Gunter et al. (2016)
		*Eurysternus caribaeus*	KU739494.1	15,227	Gunter et al. (2016)
		*Eurysternus foedus*	KU739455.1	15,366	Gunter et al. (2016)
		*Eurysternus hamaticollis*	KU739493.1	15,428	Gunter et al. (2016)
		*Eurysternus inflexus*	KU739492.1	15,766	Gunter et al. (2016)
		*Eurysternus* sp. KM‐2017	MG193385.1	15,804	Unpublished
		*Helictopleurus quadripunctatus*	KU739489.1	15,265	Gunter et al. (2016)
		*Heteronitis castelnaui*	KU739468.1	13,441	Gunter et al. (2016)
		*Liatongus bucerus*	PQ179713	15,277	This study
		*Liatongus militaris*	KU739488.1	15,832	Gunter et al. (2016)
		*Milichus apicalis*	KU739481.1	15,823	Gunter et al. (2016)
		*Oniticellus egregius*	KU739487.1	15,547	Gunter et al. (2016)
		*Onitis alexis*	KU739467.1	17,501	Gunter et al. (2016)
		*Onitis falcatus*	PQ179714	15,982	This study
		*Onitis falcatus*	KU739466.1	15,763	Gunter et al. (2016)
		*Onthophagus baolocensis*	KU739464.1	16,690	Gunter et al. (2016)
		*Onthophagus bonasus*	KU739459.1	15,730	Gunter et al. (2016)
		*Onthophagus cervicapra*	KU739449.1	14,114	Gunter et al. (2016)
		*Onthophagus cf. jeannelianus*	KU739458.1	15,654	Gunter et al. (2016)
	
		*Onthophagus cf. taurinus*	KU739462.1	15,925	Gunter et al. (2016)
		*Onthophagus crassicollis*	KU739447.1	15,370	Gunter et al. (2016)
		*Onthophagus cuniculus*	KU739451.1	15,147	Gunter et al. (2016)
		*Onthophagus diabolicus*	KU739483.1	12,098	Gunter et al. (2016)
		*Onthophagus falculatus*	KU739448.1	13,980	Gunter et al. (2016)
		*Onthophagus fimetarius*	KU739452.1	16,125	Gunter et al. (2016)
		*Onthophagus fodiens*	PQ067330.1	16,139	Unpublished
		*Onthophagus gracilipes*	KU739461.1	15,342	Gunter et al. (2016)
		*Onthophagus haematopus*	KU739478.1	15,301	Gunter et al. (2016)
		*Onthophagus longimanus*	KU739500.1	15,374	Unpublished
		*Onthophagus nitidior*	KU739499.1	14,329	Gunter et al. (2016)
		*Onthophagus nr. babirussa*	KU739480.1	15,184	Gunter et al. (2016)
		*Onthophagus obscurior*	KU739477.1	13,222	Gunter et al. (2016)
		*Onthophagus pullus*	KU739496.1	15,287	Gunter et al. (2016)
		*Onthophagus rhinolophus*	KU739498.1	15,237	Gunter et al. (2016)
		*Onthophagus rorarius*	KU739476.1	15,234	Gunter et al. (2016)
		*Onthophagus vulpes*	KU739474.1	15,884	Gunter et al. (2016)
		*Onthophagus yukae*	KU739463.1	16,362	Gunter et al. (2016)
		*Phalops ardea*	KU739473.1	16,248	Gunter et al. (2016)
		*Phalops barbicornis*	KU739457.1	13,659	Gunter et al. 2016)
		*Phalops smaragdinus*	KU739495.1	15,104	Gunter et al. (2016)
		*Proagoderus bicallossus*	KU739472.1	15,361	Unpublished
		*Proagoderus schwaneri*	KU739471.1	15,581	Unpublished
		*Scaptodera rhadamistus*	KU739460.1	15,119	Gunter et al. (2016)
		*Scarabaeidae* sp. BMNH 1274750	KT696268.1	18,626	Guo et al. (2022)
		*Scarabaeidae* sp. BMNH 1274752	KT696269.1	16,799	Guo et al. (2022)
		*Scarabaeidae* sp. BMNH 1274753	KT696270.1	16,770	Guo et al. (2022)
		*Tiniocellus sarawacus*	KU739486.1	15,592	Gunter et al. (2016)
		*Tiniocellus spinipes*	KU739485.1	15,177	Gunter et al. (2016)
*Tragiscus dimidiatus*	KU739454.1	14,206	Gunter et al. (2016)

## 3 Results

### 3.1 Mitogenome Organization and Composition

**FIGURE 1:**
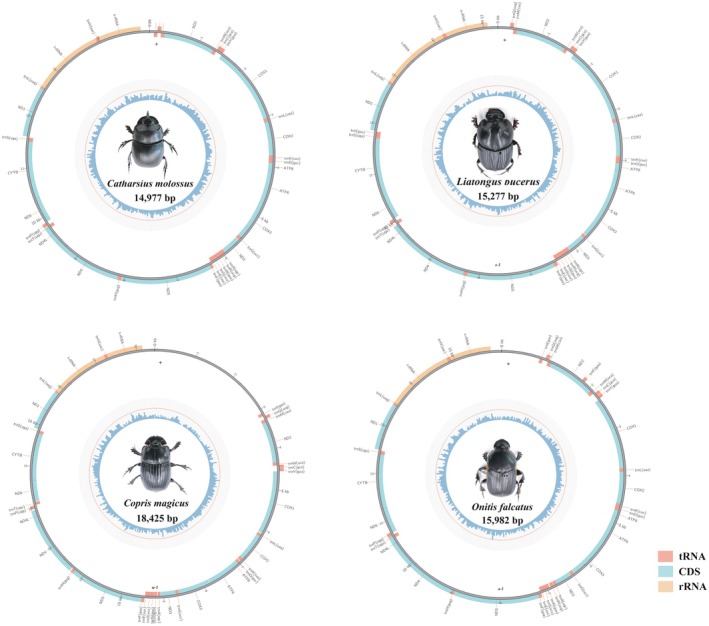
Circle map of the complete mitogenomes of four dung beetles, with different colors to distinguish different genes.

The complete mitochondrial genome structure of the four dung beetle species is depicted in Figure 1 and Appendix S1. These genomes adhere to the typical composition of insect mitochondrial genomes, comprising 37 genes in total. This includes 13 PCGs, 22 transfer RNA (tRNA) genes, and 2 ribosomal RNA (rRNA) genes. Additionally, each mitochondrial genome forms a circular DNA molecule that includes a non‐coding control region (also called “AT‐rich region”). There are 9 PCGs and 14 tRNA genes on the J‐strand, and 14 genes on the N‐strand. The complete mitochondrial genome lengths of *C. molossus* , *O. falcatus*, *C. magicus*, and *L. bucerus* were 14,977 bp, 15,982 bp, 18,425 bp, and 15,277 bp, respectively; *C. magicus* possessed the largest mitochondrial genome. Its control region is significantly longer compared to the other three species, measuring 3133 bp, 2358 bp, and 2949 bp longer than those of *C. molossus* , *O. falcatus* , and *L. bucerus* , respectively.

### 3.2 Nucleotide Composition Bias

The base composition and bias of the mitochondrial genomes of the four dung beetles are summarized in Table 2. All genomes exhibit a pronounced bias toward AT content, indicated by positive AT‐skew and negative GC‐skew values.

**TABLE 2:** Nucleotide composition of the mitochondrial genomes of four dung beetles.

Species	Whole mitochodrial genome			Protein‐coding genes			Transfer RNA genes		
	A + T (%)	AT skew	GC skew	A + T (%)	AT skew	GC skew	A + T (%)	AT skew	GC skew
*C. molossus*	77.04	0.030	−0.215	76.71	−0.153	−0.004	79.02	0.038	0.137
*L. bucerus*	78.27	0.033	−0.212	76.85	−0.132	0.004	81.75	0.019	0.190
*C. magicus*	79.80	0.045	−0.193	76.93	−0.125	0.003	80.94	0.018	0.148
*O. falcatus*	78.45	0.024	−0.161	76.90	−0.143	0.006	79.42	0.020	0.140

Species	16sRNA			12sRNA			AT‐richregion		
	A + T (%)	AT skew	GC skew	A + T (%)	AT skew	GC skew	A + T (%)	AT skew	GC skew
*C. molossus*	79.32	−0.060	0.334	73.69	−0.025	0.336	77.56	−0.036	−0.299
*L. bucerus*	81.81	−0.056	0.358	80.37	0.014	0.338	84.99	0.075	−0.334
*C. magicus*	81.64	−0.072	0.373	77.12	0.000	0.372	87.69	0.032	0.017
*O. falcatus*	82.00	−0.060	0.341	79.61	0.018	0.286	85.52	−0.025	0.073

### 3.3 Protein‐Coding Genes and Codon Usage

**FIGURE 2:**
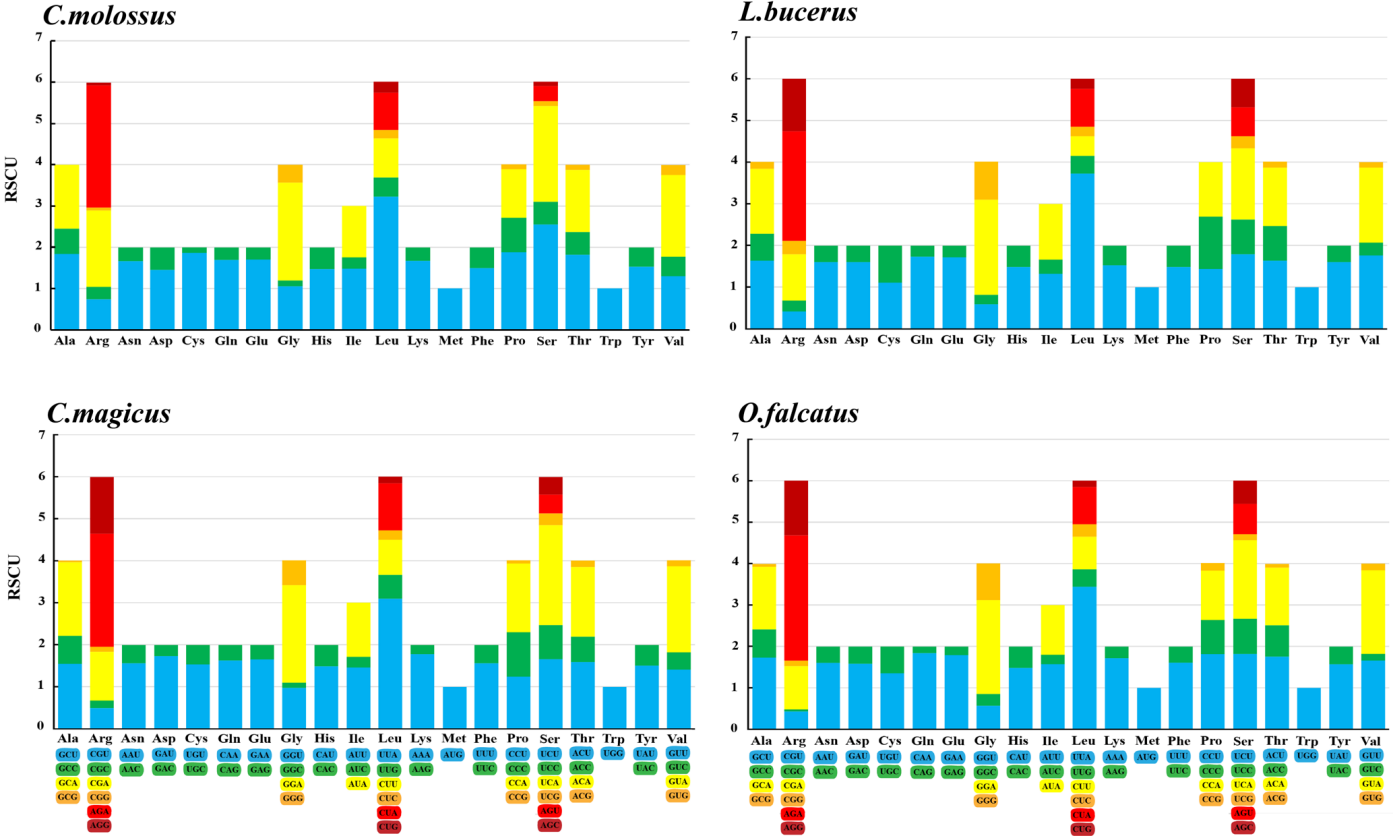
Relative synonymous codon usage (RSCU) and number of amino acids in protein‐coding genes (PCGs) of the mitogenomes of four dung beetles.

The total lengths of the 13 PCGs of *C. molossus* , *O. falcatus* , *C. magicus*, and *L. bucerus* were 11,145 bp, 11,143 bp, 11,143 bp, and 11,130 bp, respectively, with small differences. Except for the start codon of TTA for *ND5* in *O. falcatus* , the rest of the PCGs of the four dung beetles have a typical ATN/TAN(T) structure for both the start and termination codons, which is in line with the termination codons commonly used in insect mitochondrial genomes (Jeong et al. 2020; Mello et al. 2021). In the PCGs, *C. molossus* exhibited AT‐skew (−0.153) and GC‐skew (−0.004) values, suggesting a preference for the use of T and C bases. Conversely, the other three dung beetle species showed negative AT‐skew and positive GC‐skew values in their PCGs, indicating a preference for the use of T and G bases (see Table 2). In relative synonymous codon usage frequency, *C. molossus* encoded a total of 3715 amino acid residues, *O. falcatus* encoded a total of 3713 amino acid residues, *C. magicus* encoded a total of 3716 amino acid residues, and *L. bucerus* encoded a total of 3710 amino acid residues. Meanwhile, the most frequently used codons across all four dung beetle species were UUU (Phe), UUA (Leu), AUU (Ile), AUA (Ile), AAU (Asn), and AAA (Lys), with these codons being used more than 200 times (Figure 2, Appendix S1). The codon usage pattern shows a high frequency of codons composed of A and T bases. Conversely, codons rich in C and G bases generally exhibited relative synonymous codon usage (RSCU) values less than 1, indicating lower frequencies of use. This reflects a clear AT bias in the mitochondrial genomes of the four dung beetle species.

**FIGURE 3:**
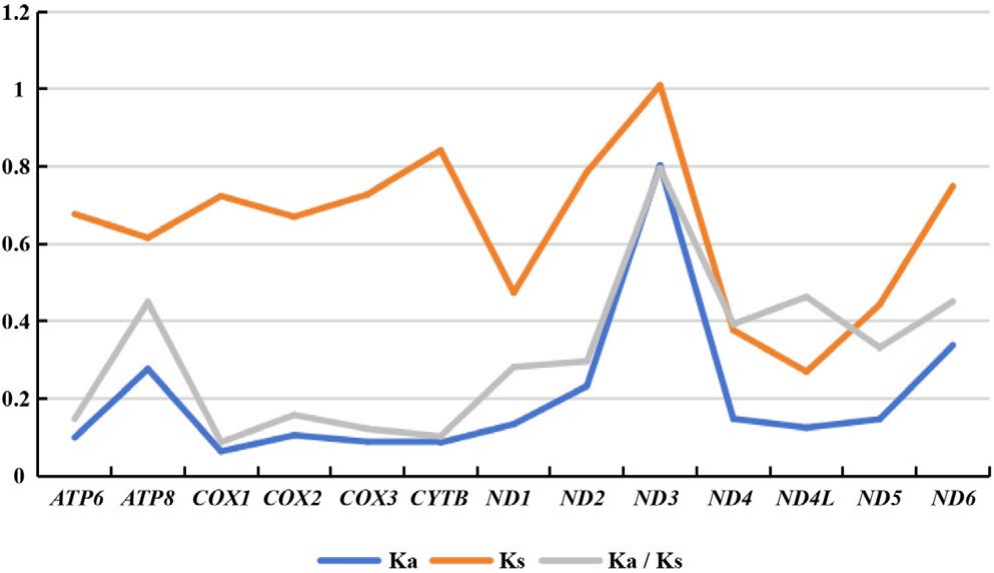
Non‐synonymous (Ka) to synonymous (Ks) substitution rates of 13 PCGs among four dung beetles.

All 13 PCGs had Ka/Ks substitution ratios less than 1, underwent purifying selection, with Ka/Ks values ranging from 0.088 (*COI*) to 0.795 (*ND3*) (Figure 3, Table 3). From this, we can see *COI*, with the smallest Ka/Ks substitution ratio among the PCGs, indicates strong conservation.

**TABLE 3:** Evolutionary rates of 13 protein‐coding genes (PCGs) of four dung beetles.

Parameters	ATP6	ATP8	COX1	COX2	COX3	CYTB	ND1	ND2	ND3	ND4	ND4L	ND5	ND6
Ka	0.100	0.277	0.064	0.106	0.089	0.087	0.134	0.233	0.803	0.148	0.125	0.147	0.338
Ks	0.677	0.616	0.724	0.670	0.728	0.842	0.474	0.786	1.010	0.377	0.270	0.443	0.749
Ka/Ks	0.148	0.450	0.088	0.158	0.122	0.103	0.282	0.297	0.795	0.393	0.463	0.332	0.451

### 3.4 tRNA and rRNA

**FIGURE 4:**
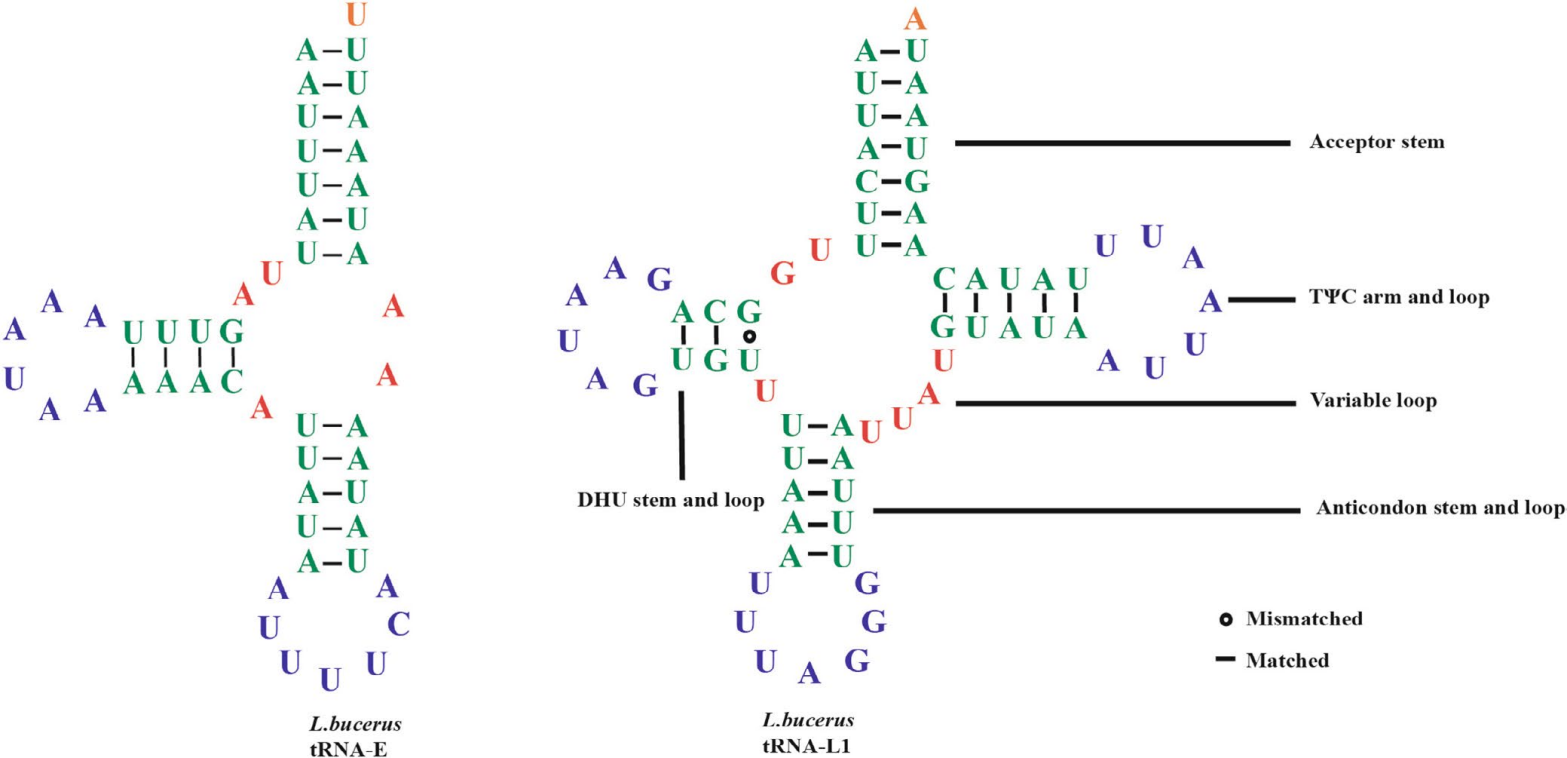
The predicted secondary structure of tRNA‐L1 and tRNA‐E in the mitogenome of *L. bucerus* .

The total lengths of the 22 tRNAs of *C. molossus* , *O. falcatus* , *C. magicus*, and *L. bucerus* were 1457 bp, 1493 bp, 1490 bp, and 1460 bp, respectively, and the gene lengths ranged from 63 to 72 bp, 65 to 72 bp, 65 to 74 bp, and 51 to 72 bp, respectively. Among the 22 tRNAs analyzed in all four dung beetles, positive AT‐skew and GC‐skew values were observed, indicating a preference for the use of A and G bases. Typically, tRNA secondary structures feature four arms: the amino acid acceptor arm, DHU arm, anticodon arm, and TΨC arm, as well as four loops: the variable loop, DHU loop, anticodon loop, and TΨC loop (Figure 4). The secondary structures of the tRNAs in these dung beetles conform to the typical cloverleaf structure, with one notable exception: the *trnE* gene in *L. bucerus* lacks a TΨC loop and TΨC stem (Appendix S1). In tRNA, there are 67 mismatched base pairs in the four dung beetles, including 17 pairs of *C. molossus* mismatch, 11 pairs of *O. falcatus* mismatch, 20 pairs of *C. magicus* mismatch, and 19 pairs of *L. bucerus* mismatch.

The total length of two rRNAs of *C. molossus* , *O. falcatus* , *C. magicus*, and *L. bucerus* was 2151 bp, 2207 bp, 2195 bp, and 2167 bp, respectively. The *rrnL* was 1349 bp, 1383 bp, 1373 bp, and 1352 bp, respectively, located between *trnL1* and *trnV*. The *rrnS* was 802 bp, 824 bp, 822 bp, and 815 bp, respectively, located between the *trnV* and control regions. In the two rRNAs, the AT‐skew values of the four dung beetles were negative, and the GC‐skew values were positive, indicating a bias towards the use of T bases and G bases.

### 3.5 Gene Overlap and Spacing

With 14, 10, 10, and 6 overlapping regions in *C. molossus* , *O. falcatus* , *C. magicus*, and *L. bucerus* , respectively, and the longest overlapping region in *C. molossus* and *C. magicus* being the overlap between *trnL1* and *rrnL*, which was 37 bp and 43 bp. Additionally, the overlap between atp6 and atp8 was observed in all four species, consistent with other insects (Zhang et al. 2014, 2021). The four dung beetle species had the longest space of 44 bp, the space between *trnI* and *trnQ* in *O. falcatus* , the space between *nad4l* and *trnP* in *C. magicus*, and the rest of the genes had spaces of less than 30 bp.

### 3.6 The Control Region (AT‐Rich Region)

**FIGURE 5:**
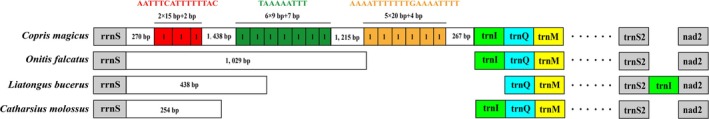
Repeat units of the control region of the mitogenome from four dung beetles. Tandem repeats were only found in *C. magicus*, which were labeled red, dark green, and orange, respectively, and the remaining white boxes represented non‐repeating regions.

The lengths of the control regions in *C. molossus* , *O. falcatus* , *C. magicus*, and *L. bucerus* were 254 bp, 1029 bp, 3387 bp, and 473 bp, respectively. These regions were located between the *rrnS* and *trnQ* genes, except for the control region in *L. bucerus* , which was situated between the *rrnS* and *trnI* genes. The structures of the control regions of the four dung beetles are depicted in Figure 5 and exhibit distinct structural differences. Specifically, the AT‐rich region of *C. magicus* features a significant tandem repeat sequence expressed in multiple copies. This region contains three distinct complete repeat units: 15 bp × 2 + 2 bp, 9 bp × 6 + 7 bp, and 20 bp × 5 + 4 bp, respectively. Tandem repeat units were identified exclusively in *C. magicus*, with no such units observed in the control regions of the other three dung beetles. These tandem repeats consist of three repetitive sequences with lengths of 26 bp, 29 bp, and 21 bp, respectively, within the control region.

### 3.7 Gene Rearrangement

Two types of gene rearrangements were identified: (1) position swapping of *trnT* and *trnP* genes in *C. magicus*, forming a *trnP‐trnT* gene block; (2) long‐distance translocation of the *trnI* gene between *trnS2* and *nad1* in *L. bucerus* , creating a *trnS2‐trnI* gene block.

### 3.8 Phylogenetic Analyses

**FIGURE 6:**
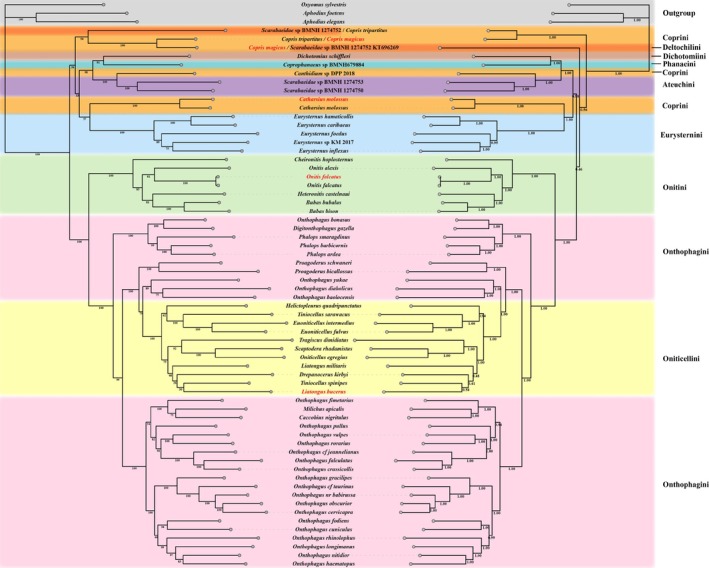
Phylogenetic relationship of Scarabaeinae based on nucleotide sequences of 13 protein‐coding genes in the mitogenome. The left part of the clade represents the maximum likelihood construct and its support value, and the right part represents the Bayesian construct and its Bayesian posterior probability value. The species name in red colors were sequenced in this study.

The optimal substitution model for phylogenetic tree construction was determined to be JC + I + G, and the phylogenetic trees were constructed (Figure 6). The ML tree exhibits a topology highly like that of the BI tree, with strong support at most branch nodes in both phylogenetic trees. Phylogenetic relationships of the nine Scarabaeinae tribes are as follows: the Coprini tribe demonstrates polyphyly, appearing in clades closely affiliated with the tribes Deltochilini, Dichotomiini, Phanaeini, Ateuchini, and Eurysternini; the Onitini tribe is closest to the Onthophagini tribe; and the Onthophagini tribe displays polyphyly, being in a sister group relationship with the Oniticellini tribe. Among them, the tribes Onitini and Oniticellini showed monophyly, while the tribes Coprini and Onthophagini exhibited polyphyly. The four dung beetles used in this study except *L. bucerus* clustered under the same branch with species of the same genus, supporting the morphological classification results. Within the Onthophagini tribe, *Digitonthophagus gazella*, *Milichus apicalis*, and *Caccobius nigritulus* clustered robustly within the clade containing species of the type genus *Onthophagus* (based the standard BS > 69 or PP > 0.89 (Cai et al. 2019)), consistent with current classification. In general, the phylogenetic relationships between the tribes in this study can be represented as CopriniI + (Deltochilini + ((((Dichotomiini + Phanaeini) + (CopriniII + Ateuchini)) + (CopriniIII + Eurysternini))) + (Onitini + (OnthophaginiI + (Oniticellini + OnthophaginiII)))).

## 4 Discussion

### 4.1 Mitochondrial Genome Characterization

The total mitochondrial genome length is often affected by the length of the control region in all four dung beetle species. It is consistent with the general understanding in eukaryotes (Ayivi et al. 2021). There is a general preference for A and T bases over G and C bases in insect mitochondrial genomes (Cameron 2014; Song and Zhang 2018). The AT‐rich codon usage in all four dung beetle species is consistent with the overall base composition bias of insect mitochondrial genomes; however, variations in base preference at different positions are observed among the species. For example, in the control regions, all four dung beetle species exhibited distinct base preferences, with *C. molossus* favoring T and C bases, *O. falcatus* favoring T and G, *C. magicus* favoring A and G, and *L. bucerus* favoring A and C. The nucleotide composition bias phenomenon in insects has received long‐term attention. So far, this is generally attributed to factors such as a combination of mutational pressure favoring A/T substitutions, reduced constraints on codon usage in mitochondrial genes, and potential selection for replication or transcriptional efficiency (Jeong et al. 2020; Mello et al. 2021; Cameron 2014). We also found that the top three most frequently used amino acids of the four dung beetles are consistent with the species *Anomala corpulenta* from the Rutelinae subfamily (Qu et al. 2023) and with the species *Simulium ornatum* in Diptera (Sun et al. 2023). This is indeed a question that is confusing and worthy of further research.

*COI* exhibited the lowest Ka/Ks ratio among the PCGs of the four dung beetles, indicating that it is under strong purifying selection. It reflects the stringent functional constraints acting on *COI*, and it is likely due to its essential role in mitochondrial function and energy production. Meanwhile, the high sequence conservation of *COI* across the four dung beetle species suggests it is less tolerant to genetic variation, which could be for reinforcing its functional indispensability (Yang et al. 2018). Such conservation patterns also emphasize the heterogeneous evolutionary dynamics among mitochondrial genes. While *COI* may not directly promote genetic diversity, its stability under selective pressure might provide a reliable molecular marker for phylogenetic reconstruction in Scarabaeinae.

The secondary structures of the tRNAs in the four dung beetles conform to the typical cloverleaf structure, with one notable exception: the *trnE* gene in *L. bucerus* lacks a TΨC loop and TΨC stem. The TΨC loop and TΨC stem play important roles in the normal folding and function of tRNAs, and atypical secondary structures may hinder the function of tRNAs (Shimodaira 2001). So, *L. bucerus* may have some differences in the functional expression of this gene. In tRNA, there are 67 mismatched base pairs in the four dung beetles, including 17 pairs of *C. molossus* mismatch, 11 pairs of *O. falcatus* mismatch, 20 pairs of *C. magicus* mismatch, and 19 pairs of *L. bucerus* mismatch. The mismatch type is G‐U, and G‐U mismatch is very common in the insect mitochondrial genome, which may be caused by a small number of base pairing types (Hu et al. 2020).

### 4.2 Two New Types of Gene Rearrangement

Gene rearrangements in insect mitochondria serve as valuable phylogenetic signals for identifying rearrangement patterns across lineages and uncovering shared traits. Specific gene sequences resulted by gene rearrangements are crucial markers for delineating taxonomic boundaries and analyzing evolutionary relationships among species. These markers enhance higher‐order phylogenetic analyses, thereby increasing the reliability of phylogenetic outcomes (Cameron 2014; Dowton et al. 2002; Negrisolo et al. 2011). Among reported insects, gene rearrangements in Coleoptera are rarest, where the order of gene arrangement typically follows the ancestral pattern observed in insects (Yu et al. 2023; Andujar et al. 2017). As of April 1, 2024, all mitochondrial whole genomes within Scarabaeinae were extracted from the NCBI database for this study. No gene rearrangements other than *trnT‐trnP* were found in the species within Scarabaeinae. The *trnI‐trnQ‐trnM* gene block is a hotspot for gene rearrangements in insect mitochondrial genomes (Ayivi et al. 2021), exemplified by the *trnQ‐trnI‐trnM* gene cluster found in species of Dynastinae (Ayivi et al. 2021; Dowton and Austin 1999; Cheng et al. 2021; Filipovic et al. 2021). Although gene rearrangements in insects are primarily dominated by the *trnI‐trnQ‐trnM* arrangement, and this leads to two other types of rearrangements: *trnT‐trnP* and *trnS2‐trnI*. The “tandem duplication, random loss” (TDRL) model hypothesized that the *trnT‐trnP* rearrangement might occur through tandem duplication of the *trnT‐trnP* gene block. This process could lead to random loss of *trnT* in the first copy of the *trnT‐trnP* region and random loss of *trnP* in the second copy (Cameron 2014). The *trnS2‐trnI* rearrangement identified in *L. bucerus* represents the first instance of such rearrangement in Scarabaeidae. Random loss following tandem duplication could potentially be a mechanism contributing to this long‐distance translocation. Although this possibility is very low.

Beyond the TDRL, factors involved in relationships with parasitism could influence gene rearrangements. In habitats for dung beetles, such as pastures, mites (acari), nematodes, and other organisms are abundant, exhibiting intricate interactions with the beetles (Ari et al. 2022; Boze and Moore 2014). These organisms may disrupt the reproductive cycles of dung beetles, influence their genetic processes, and even lead to gene rearrangements. These factors, such as AT content and evolutionary rates, also could collectively contribute to the emergence of gene recombinations (Dowton et al. 2002).

As observed in the current study, the gene rearrangements exclusively affected tRNA genes. *C. magicus* shares an identical gene arrangement with its genus counterpart *C. tripartitus* (Jeong et al. 2020) and *Onthophagus yukae* (GB: KU739463) within the same subfamily. In contrast, *L. bucerus* displayed a gene rearrangement not observed in its genus‐mate, *L. militaris* (GB: KU739488.1). It indicates that mitochondrial gene orders can vary among different species within the same genus (Shao and Barker 2003; Liu et al. 2017). A The novel gene arrangement pattern has so far been identified only in *Copris*. It seems to be due to the number of reported mitochondrial genomes in this subfamily remaining very limited. The *trnT‐trnP* rearrangement is currently observed solely in *O. yukae* (genus *Onthophagus*). This gene rearrangement may be homoplastic; more of these rearrangements may be found in this genus or in Scarabaeinae.

### 4.3 Phylogenetic Analyses of Scarabaeinae

Overall, the phylogenetic topology tree obtained in this study is consistent with previous results, for example, studies support Onthophagini as a multilineage group, which is consistent with the findings of (Tarasov and Génier 2015). However, some dubious phylogenetic relationships between species of different tribes appeared.

Firstly, although *Dichotomius schiffleri* and *Coprophanaeus* sp. belong to different tribes, they are clustered together in the current research. However, it does not seem to be a bad thing and may provide clues to the polyphyly of the Coprini tribe. In the history of taxonomy, Olsoufieff (1924) and Sturm (1826) once classified the *Coprophanaeus* genus (it belongs to the Phanacini tribe nowdays) within the tribe Coprini based on adult morphological characters. *Coprophanaeus* sp., inserted into the topological structure of the polyphyletic Coprini tribe, maintained a close affinity with Coprini. Vaz‐de‐Mello et al. (2001) classified *Dichotomius* as the type genus of the tribe Dichotomiini, which has a stable taxonomic history. In the present study, *Dichotomius schiffleri* was found to be sister to *Coprophanaeus* sp. with support values of BS = 1 and PP = 91. Beyond the possibility of species identification errors, it is highly probable that the tribe Phanaeini and Coprini are related to each other as sister groups in terms of taxonomic status, which agrees with the findings of (Mello et al. 2021). As early as 1801, Fabricius (1801) placed *C. molossus* within *Copris*, and later Hope (1837) classified it under *Catharsius*, suggesting that *C. molossus* has historically been considered part of the Coprini taxon. *C. molossus* and *C. molossus* uploaded in the NCBI database can be clustered into the same clade, but the genetic distance is relatively distant, indicating that the same species can have obvious genetic differentiation under the influence of external conditions. In this case, *Dichotomius schiffleri* and *Coprophanaeus* sp., who are also in the same branch, seem to have more stories to explore. Therefore, it is worth further studying whether the Coprini tribe is truly a polyphyly and its relationship with the tribes Deltochilini, Dichotomiini, Phanaeini, and Ateuchini.

Secondly, the type genus *Onthophagus* species of Onthophagini did n't all gather into one evolutionary branch. It seems to require more work, whether in morphology or molecular biology, to address the relationship between *Onthophagus bonasus*, *O. diabolicus* , *O. baolocensis*, and branches of the *Onthophagus* genus, as the three species are classified into different branches. Interestingly, *Digitonthophagus gazella*, *Milichus apicalis*, and *Caccobius nigritulus* have been placed within the *Onthophagus* branch, and their taxonomic history shows that they were previously classified under *Onthophagus* (Klug 1855; Reiche 1840; Hraeus 1857).

Thirdly, the tribes Onitini and Oniticellini exhibited monophyly, consistent with the previous findings (Tarasov and Génier 2015; Philips et al. 2004; Villalba et al. 2002). However, the taxonomic relationships among genera within Onitini and Oniticellini are complex. For instance, *Tiniocellus spinipes*, *D. kirbyi* , and species of *Liatongus* are clustered together in a single group, while *Cheironitis hoplosternus* is clustered together with species of *Onitis* (Roth 1851). Their taxonomic history reveals that *T. spinipes* , *D. kirbyi* , *L. militaris* , and *L. bucerus* were previously classified under *Oniticellus* (Roth 1851; Castelnau 1840). It suggests that these dung beetles inherently share many similar features in their morphological classification. However, why do neither the BS nor the PP values support their clustering relationship? The stability of the phylogenetic tree relies on features; the lack of sufficient genes or data features may result in lower support values. A wider range of species and more nucleic acid information are needed.

A significant source of confusion in the classification of Scarabaeinae tribes and genera seems to stem from the predominant use of morphological characters by earlier research. Besides, the limited application of molecular techniques, or the use of shorter gene fragments, poses challenges in distinguishing between species. Therefore, the acquisition of more molecular data from both nuclear and mitochondrial genes will facilitate a clearer understanding of the phylogenetic relationships among dung beetle species.

## 5 Conclusion

We sequenced and analyzed the complete mitochondrial genomes of *C. molossus* , *L. bucerus* , *C. magicus*, and *O. falcatus* from the Scarabaeinae subfamily, and compared the structural features of these genomes with sequences from 47 other Scarabaeinae species. Additionally, we employed ML and BI methods to construct a phylogenetic tree based on the mitochondrial genomes.

1. The complete mitochondrial genomes of *C. molossus* , *O. falcatus* , *C*. *magicus*, and *L. bucerus* were 14,977 bp, 15,982 bp, 18,425 bp, and 15,277 bp in length, respectively, which were consistent with the structure of insect mitochondrial genomes, showing an obvious AT bias. The tRNA secondary structures adhered to typical cloverleaf structures, except for the absence of a TΨC loop and TΨC stem in the *trnE* gene of *L. bucerus* . The mitochondrial genomes of *L. bucerus* and *C. magicus* exhibit high conservation. However, two types of gene rearrangements were observed in two other dung beetles: in *C. magicus*, the *trnT* and *trnP* genes swapped positions, forming a *trnP‐trnT* tRNA gene block; in *L. bucerus* , the *trnI* gene underwent a long‐distance translocation and rearranged between the *trnS2* and *nad1* genes, forming a *trnS2‐trnI* tRNA gene block. Among the PCGs of the four dung beetle species, all 13 PCGs showed purifying selection, with *COI* evolving at the slowest rate.
2. The ML and BI phylogenetic trees constructed exhibit similar topological structures with strong support. The polyphyly of the tribes Coprini and Onthophagini is supported, and Coprini maintained close affinities with Deltochilini, Dichotomiini, Phanaeini, Ateuchini, Phanaeini, and Eurysternini. The tribes Onitini and Oniticellini are monophyletic. The phylogenetic relationship among the tribes in this study should be CopriniI + (Deltochilini + ((((Dichotomiini + Phanaeini) + (CopriniII + Ateuchini)) + (CopriniIII + Eurysternini))) + (Onitini + (OnthophaginiI + (Oniticellini + OnthophaginiII)))). Combined with the taxonomic history of the species, the results clarified the position of the sequenced dung beetles in their respective tribes and genera.

This study enriches the mitogenomic data for Scarabaeinae, confirming the utility of mitochondrial genomes in resolving dung beetle phylogeny and providing a foundation for elucidating evolutionary relationships within this subfamily.

## Author Contributions

**Honghui Zhang:** formal analysis (equal), investigation (equal), methodology (equal), validation (equal), visualization (lead), writing – original draft (equal). **Qiuju He:** data curation (equal), formal analysis (equal), writing – original draft (equal). **Zhiyong Zhao:** investigation (equal), resources (equal). **Bin Zhang:** investigation (equal), resources (equal). **Jun Zhou:** investigation (equal), software (equal), validation (supporting). **Chengye Wang:** formal analysis (equal), methodology (equal), software (equal). **Chuanhui Yi:** conceptualization (equal), methodology (equal), writing – review and editing (equal). **Min Zhao:** conceptualization (lead), funding acquisition (lead), methodology (equal), project administration (lead), resources (equal), supervision (lead), writing – review and editing (equal).

## Conflicts of Interest

The authors declare no conflicts of interest.

## Supporting information


**Appendix S1:** ece371906‐sup‐0001‐Appendix.docx.

## Data Availability

Sequence data that support the findings of this study have been deposited in the NCBI (https://www.ncbi. nlm.nih.gov/) GenBank database with the primary accession numbers PQ179711, PQ179712, PQ179713, and PQ179714.
