# Supplementary material for: Mitochondrial Genome Comparison and Phylogenetic Analysis of Four Species of Dung Beetles (Coleoptera: Scarabaeidae: Scarabaeinae)
Source: Ecol Evol. 2025 Aug 5;15(8):e71906. doi: 10.1002/ece3.71906 (PMC12322740; doi:10.1002/ece3.71906)
Supplement: Supplementary file 1 — Appendix S1: ece371906‐sup‐0001‐Appendix.docx. [file ECE3-15-e71906-s001.docx]

**Appendices**

Attachment A. The predicted secondary structure of tRNA in the *Catharsius molossus*
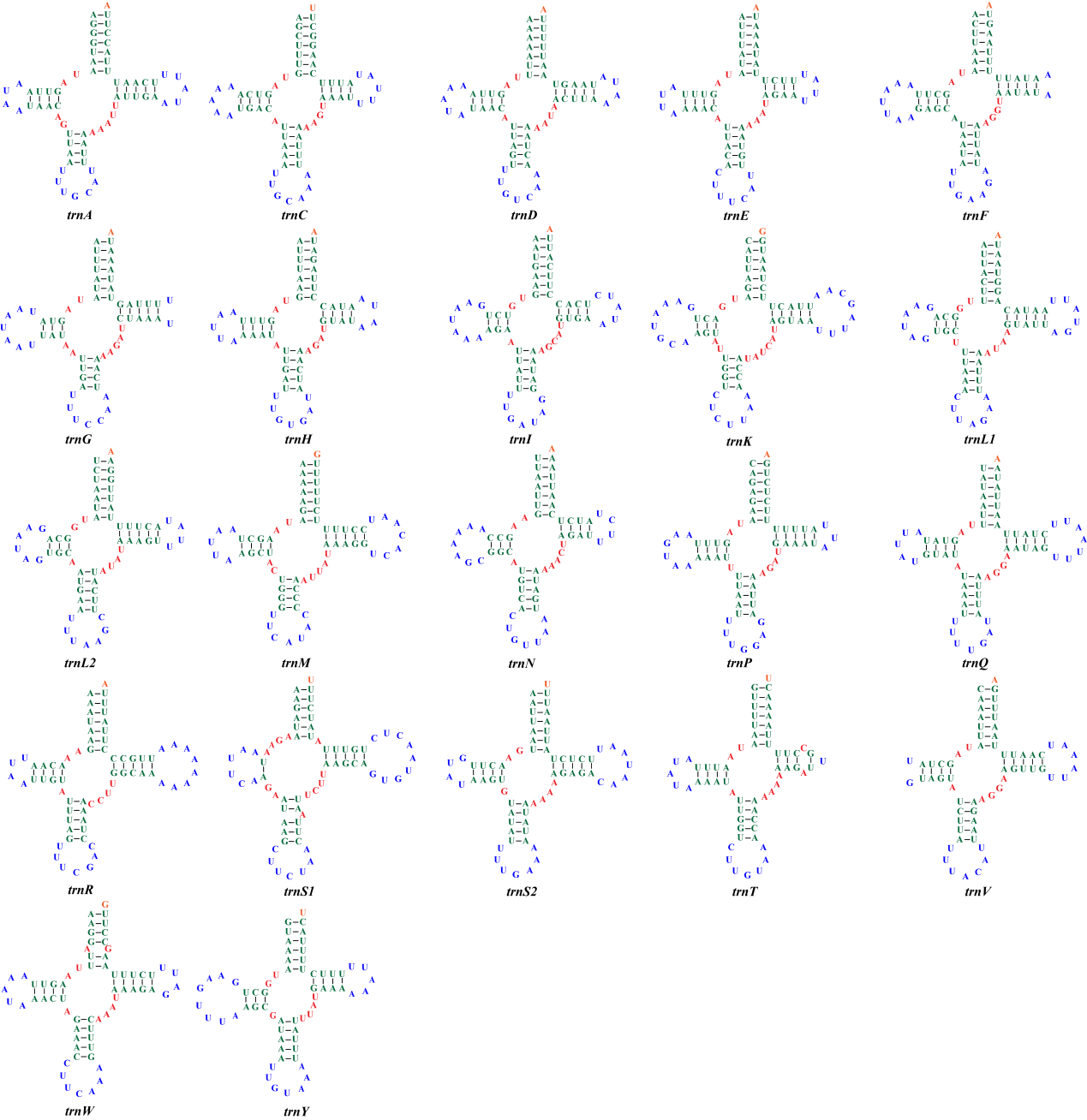


Attachment B. The predicted secondary structure of tRNA in the *Copris magicus*

*
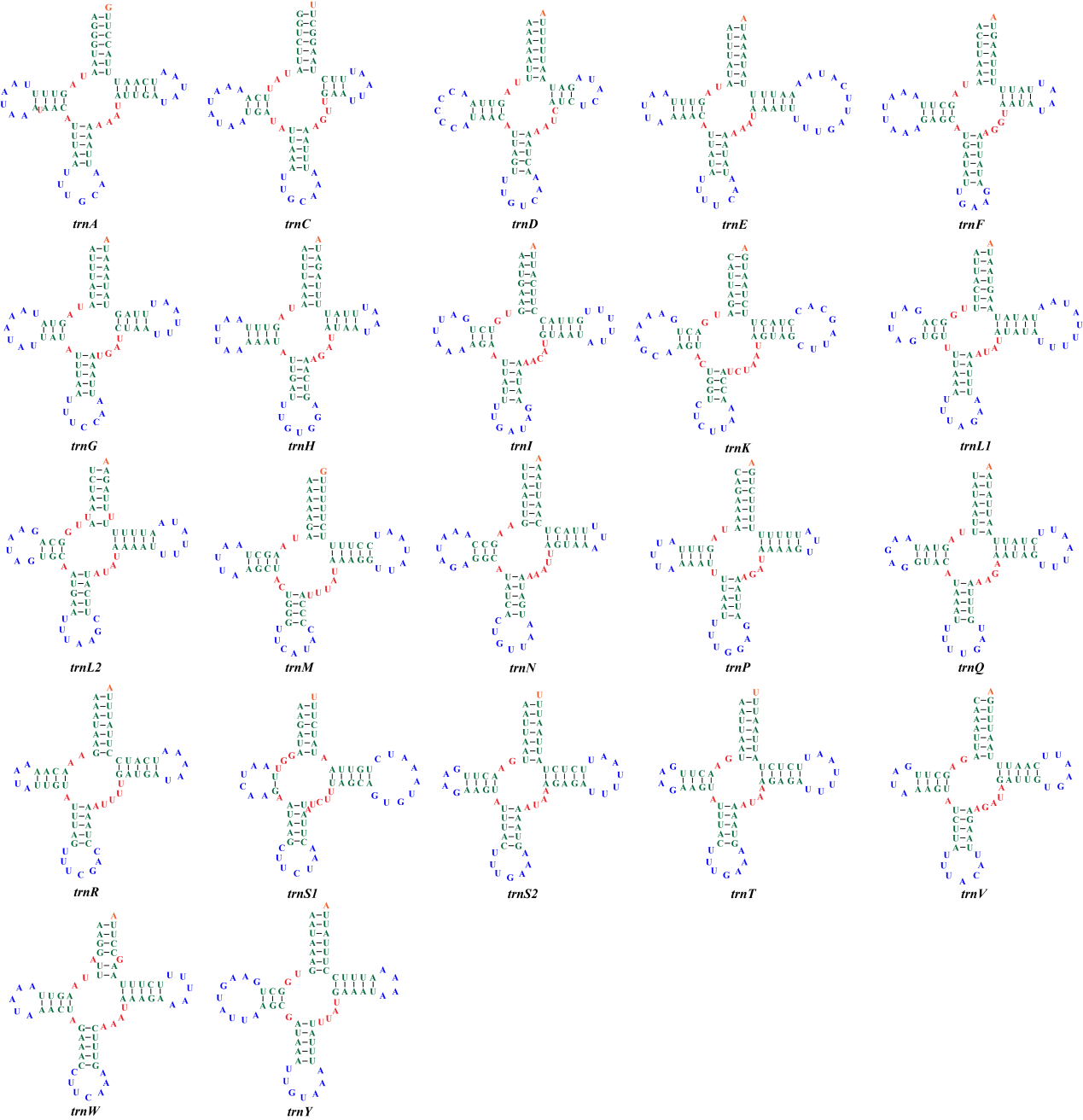
*

Attachment C. The predicted secondary structure of tRNA in the *Liatongus bucerus*

*
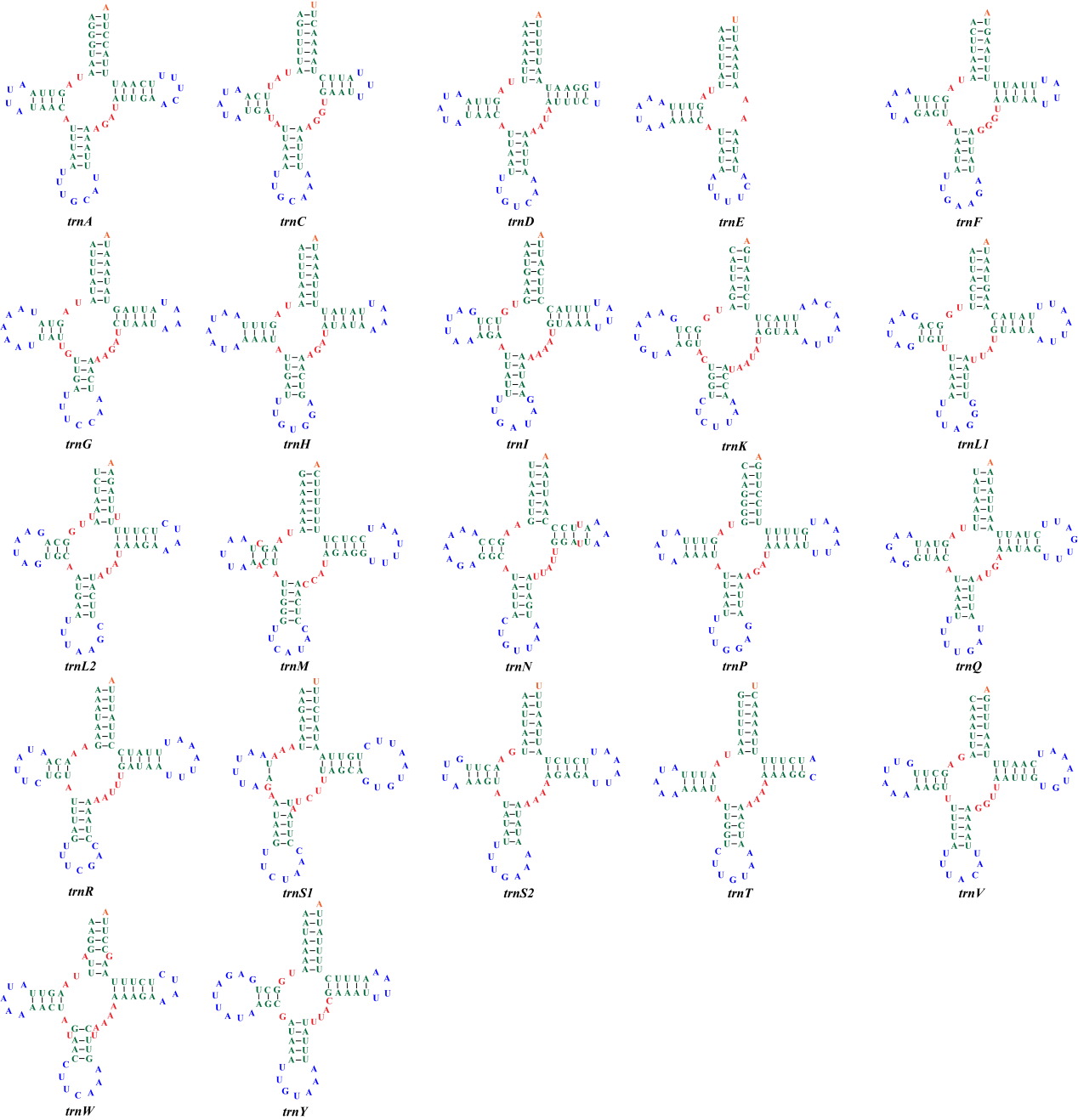
*

Attachment D. The predicted secondary structure of tRNA in the *Onitis falcatus*

*
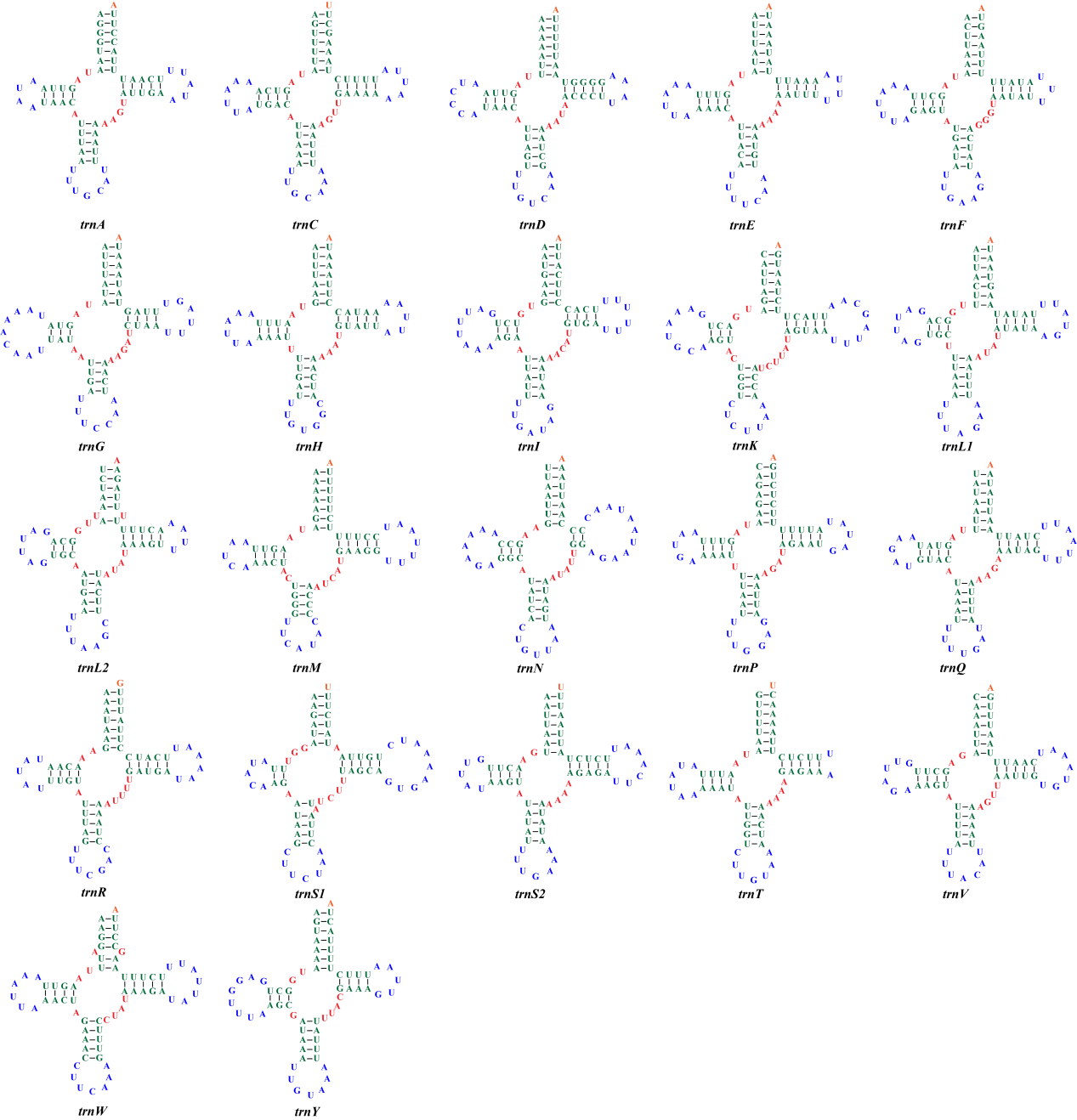
*

Schedule

A. Mitochondrial genome composition of *Catharsius molossus*

| Gene | Start Position/bp | Stop Position/bp | Coding strand | Length/bp | Start /Stop codon | Anticodon | Intergenic length/bp |
| --- | --- | --- | --- | --- | --- | --- | --- |
|  |  |  |  |  |  |  |  |
| trnI(gat) | 87 | 152 | + | 66 |  | GAT | 2 |
| trnQ(ttg) | 155 | 223 | - | 69 |  | TTG | -1 |
| trnM(cat) | 223 | 291 | + | 69 |  | CAT | 0 |
| nad2 | 292 | 1302 | + | 1011 | ATT/TAA |  | -2 |
| trnW(tca) | 1301 | 1366 | + | 66 |  | TCA | -8 |
| trnC(gca) | 1359 | 1423 | - | 65 |  | GCA | 0 |
| trnY(gta) | 1424 | 1488 | - | 65 |  | GTA | 3 |
| cox1 | 1492 | 3030 | + | 1539 | ATG/TAA |  | 2 |
| trnL2(taa) | 3033 | 3097 | + | 65 |  | TAA | 0 |
| cox2 | 3098 | 3784 | + | 687 | ATA/T |  | -8 |
| trnK(ctt) | 3777 | 3848 | + | 72 |  | CTT | 0 |
| trnD(gtc) | 3849 | 3914 | + | 66 |  | GTC | 0 |
| atp8 | 3915 | 4070 | + | 156 | ATT/TAA |  | -7 |
| atp6 | 4064 | 4738 | + | 675 | ATG/TAA |  | -1 |
| cox3 | 4738 | 5522 | + | 785 | ATG/TA |  | -1 |
| trnG(tcc) | 5522 | 5585 | + | 64 |  | TCC | 9 |
| nad3 | 5595 | 5939 | + | 345 | ATT/TAA |  | 8 |
| trnA(tgc) | 5948 | 6012 | + | 65 |  | TGC | 0 |
| trnR(tcg) | 6013 | 6081 | + | 69 |  | TCG | 2 |
| trnN(gtt) | 6084 | 6148 | + | 65 |  | GTT | 0 |
| trnS1(tct) | 6149 | 6217 | + | 69 |  | TCT | 0 |
| trnE(ttc) | 6218 | 6280 | + | 63 |  | TTC | 5 |
| trnF(gaa) | 6286 | 6351 | - | 66 |  | GAA | -5 |
| nad5 | 6347 | 8065 | - | 1719 | ATT/TAA |  | 0 |
| trnH(gtg) | 8066 | 8129 | - | 64 |  | GTG | -1 |
| nad4 | 8129 | 9466 | - | 1338 | ATG/TAA |  | -7 |
| nad4l | 9460 | 9750 | - | 291 | ATG/TAA |  | 2 |
| trnT(tgt) | 9753 | 9815 | + | 63 |  | TGT | 0 |
| trnP(tgg) | 9816 | 9881 | - | 66 |  | TGG | 11 |
| nad6 | 9893 | 10384 | + | 492 | ATT/TAA |  | -1 |
| cob | 10384 | 11520 | + | 1137 | ATG/TAA |  | 3 |
| trnS2(tga) | 11524 | 11591 | + | 68 |  | TGA | 24 |
| nad1 | 11616 | 12566 | - | 951 | ATT/TAG |  | 0 |
| trnL1(tag) | 12567 | 12633 | - | 67 |  | TAG | -37 |
| rrnL | 12597 | 13945 | - | 1349 |  |  | -2 |
| trnV(tac) | 13944 | 14008 | - | 65 |  | TAC | -1 |
| rrnS | 14008 | 14809 | - | 802 |  |  | 0 |
| A+T rich region | 14810 | 86 |  | 254 |  |  |  |

B. Mitochondrial genome composition of *Liatongus bucerus*

| Gene | Start Position/bp | Stop Position/bp | Coding strand | Length/bp | Start /Stop codon | Anticodon | Intergenic length/bp |
| --- | --- | --- | --- | --- | --- | --- | --- |
|  |  |  |  |  |  |  |  |
| trnQ(ttg) | 241 | 309 | - | 69 |  | TTG | 2 |
| trnM(cat) | 312 | 380 | + | 69 |  | CAT | 0 |
| nad2 | 381 | 1394 | + | 1014 | ATT/TAA |  | 6 |
| trnW(tca) | 1401 | 1466 | + | 66 |  | TCA | 25 |
| trnC(gca) | 1492 | 1554 | - | 63 |  | GCA | 0 |
| trnY(gta) | 1555 | 1622 | - | 68 |  | GTA | 2 |
| cox1 | 1625 | 3166 | + | 1542 | ATT/TAA |  | 3 |
| trnL2(taa) | 3170 | 3234 | + | 65 |  | TAA | 0 |
| cox2 | 3235 | 3918 | + | 684 | ATA/TAA |  | 7 |
| trnK(ctt) | 3926 | 3997 | + | 72 |  | CTT | 1 |
| trnD(gtc) | 3999 | 4062 | + | 64 |  | GTC | 0 |
| atp8 | 4063 | 4218 | + | 156 | ATA/TAA |  | -7 |
| atp6 | 4212 | 4886 | + | 675 | ATG/TAA |  | -1 |
| cox3 | 4886 | 5671 | + | 786 | ATG/TAA |  | 13 |
| trnG(tcc) | 5685 | 5750 | + | 66 |  | TCC | 9 |
| nad3 | 5760 | 6104 | + | 345 | ATT/TAA |  | 9 |
| trnA(tgc) | 6114 | 6179 | + | 66 |  | TGC | 0 |
| trnR(tcg) | 6180 | 6248 | + | 69 |  | TCG | 3 |
| trnN(gtt) | 6252 | 6317 | + | 66 |  | GTT | 0 |
| trnS1(tct) | 6318 | 6386 | + | 69 |  | TCT | 4 |
| trnE(ttc) | 6391 | 6441 | + | 51 |  | TTC | 12 |
| trnF(gaa) | 6454 | 6519 | - | 66 |  | GAA | -1 |
| nad5 | 6519 | 8231 | - | 1713 | ATT/TAA |  | 0 |
| trnH(gtg) | 8232 | 8299 | - | 68 |  | GTG | 2 |
| nad4 | 8302 | 9636 | - | 1335 | ATG/TAA |  | -7 |
| nad4l | 9630 | 9920 | - | 291 | ATG/TAA |  | 4 |
| trnT(tgt) | 9925 | 9988 | + | 64 |  | TGT | 0 |
| trnP(tgg) | 9989 | 10056 | - | 68 |  | TGG | 2 |
| nad6 | 10059 | 10553 | + | 495 | ATT/TAA |  | 3 |
| cob | 10557 | 11699 | + | 1143 | ATG/TAA |  | 10 |
| trnS2(tga) | 11710 | 11776 | + | 67 |  | TGA | 2 |
| trnI(gat) | 11779 | 11844 | + | 66 |  | GAT | 18 |
| nad1 | 11863 | 12813 | - | 951 | ATT/TAG |  | 0 |
| trnL1(tag) | 12814 | 12881 | - | 68 |  | TAG | -36 |
| rrnL | 12846 | 14197 | - | 1352 |  |  | -2 |
| trnV(tac) | 14196 | 14265 | - | 70 |  | TAC | -1 |
| rrnS | 14265 | 15079 | - | 815 |  |  | 0 |
| A+T rich region | 15080 | 240 |  | 438 |  |  |  |

C. Mitochondrial genome composition of *Copris magicus*

| Gene | Start Position/bp | Stop Position/bp | Coding strand | Length/bp | Start /Stop codon | Anticodon | Intergenic length/bp |
| --- | --- | --- | --- | --- | --- | --- | --- |
|  |  |  |  |  |  |  |  |
| trnI(gat) | 3101 | 3169 | + | 69 |  | GAT | 24 |
| trnQ(ttg) | 3194 | 3262 | - | 69 |  | TTG | -1 |
| trnM(cat) | 3262 | 3330 | + | 69 |  | CAT | 3 |
| nad2 | 3334 | 4344 | + | 1011 | ATC/TAA |  | -2 |
| trnW(tca) | 4343 | 4409 | + | 67 |  | TCA | -8 |
| trnC(gca) | 4402 | 4467 | - | 66 |  | GCA | 11 |
| trnY(gta) | 4479 | 4546 | - | 68 |  | GTA | 7 |
| cox1 | 4554 | 6089 | + | 1536 | ATG/TAA |  | 24 |
| trnL2(taa) | 6114 | 6180 | + | 67 |  | TAA | 0 |
| cox2 | 6181 | 6868 | + | 688 | ATA/T |  | -6 |
| trnK(ctt) | 6863 | 6934 | + | 72 |  | CTT | 17 |
| trnD(gtc) | 6952 | 7016 | + | 65 |  | GTC | 0 |
| atp8 | 7017 | 7172 | + | 156 | ATT/TAA |  | -7 |
| atp6 | 7166 | 7837 | + | 672 | ATG/TAA |  | 11 |
| cox3 | 7849 | 8634 | + | 786 | ATG/TAA |  | 3 |
| trnG(tcc) | 8638 | 8703 | + | 66 |  | TCC | -3 |
| nad3 | 8701 | 9057 | + | 357 | ATA/TAA |  | 13 |
| trnA(tgc) | 9071 | 9139 | + | 69 |  | TGC | 20 |
| trnR(tcg) | 9160 | 9226 | + | 67 |  | TCG | 5 |
| trnN(gtt) | 9232 | 9297 | + | 66 |  | GTT | 0 |
| trnS1(tct) | 9298 | 9364 | + | 67 |  | TCT | 3 |
| trnE(ttc) | 9368 | 9441 | + | 74 |  | TTC | 13 |
| trnF(gaa) | 9455 | 9521 | - | 67 |  | GAA | -1 |
| nad5 | 9521 | 11233 | - | 1713 | ATT/TAA |  | 0 |
| trnH(gtg) | 11234 | 11298 | - | 65 |  | GTG | 30 |
| nad4 | 11329 | 12666 | - | 1338 | ATG/TAA |  | -7 |
| nad4l | 12660 | 12950 | - | 291 | ATG/TAA |  | 44 |
| trnP(tgg) | 12995 | 13059 | - | 65 |  | TGG | 11 |
| trnT(tgt) | 13071 | 13136 | + | 66 |  | TGT | 17 |
| nad6 | 13154 | 13654 | + | 501 | ATT/TAA |  | 2 |
| cob | 13657 | 14799 | + | 1143 | ATG/TAA |  | 5 |
| trnS2(tga) | 14805 | 14872 | + | 68 |  | TGA | 22 |
| nad1 | 14895 | 15845 | - | 951 | ATT/TAG |  | 0 |
| trnL1(tag) | 15846 | 15915 | - | 70 |  | TAG | -43 |
| rrnL | 15873 | 17245 | - | 1373 |  |  | -3 |
| trnV(tac) | 17243 | 17310 | - | 68 |  | TAC | 6 |
| rrnS | 17317 | 18138 | - | 822 |  |  | 0 |
| A+T rich region | 18139 | 3100 |  | 3387 |  |  |  |

D. Mitochondrial genome composition of *Onitis falcatus*

| Gene | Start Position/bp | Stop Position/bp | Coding strand | Length/bp | Start /Stop codon | Anticodon | Intergenic length/bp |
| --- | --- | --- | --- | --- | --- | --- | --- |
|  |  |  |  |  |  |  |  |
| trnI(gat) | 1 | 68 | + | 68 |  | GAT | 44 |
| trnQ(ttg) | 113 | 181 | - | 69 |  | TTG | 1 |
| trnM(cat) | 183 | 251 | + | 69 |  | CAT | 0 |
| nad2 | 252 | 1268 | + | 1017 | ATT/TAA |  | -1 |
| trnW(tca) | 1268 | 1337 | + | 70 |  | TCA | 17 |
| trnC(gca) | 1355 | 1422 | - | 68 |  |  | 10 |
| trnY(gta) | 1433 | 1498 | - | 66 |  | GCA | 13 |
| cox1 | 1512 | 3049 | + | 1538 | ATG/T |  | -1 |
| trnL2(taa) | 3049 | 3113 | + | 65 |  | TAA | 0 |
| cox2 | 3114 | 3795 | + | 682 | ATA/T |  | -3 |
| trnK(ctt) | 3793 | 3864 | + | 72 |  | CTT | 2 |
| trnD(gtc) | 3867 | 3933 | + | 67 |  | GTC | 0 |
| atp8 | 3934 | 4089 | + | 156 | ATT/TAA |  | -7 |
| atp6 | 4083 | 4757 | + | 675 | ATG/TAA |  | 2 |
| cox3 | 4760 | 5545 | + | 786 | ATG/TAA |  | 11 |
| trnG(tcc) | 5557 | 5624 | + | 68 |  | TCC | -3 |
| nad3 | 5622 | 5978 | + | 357 | ATA/TAA |  | 0 |
| trnA(tgc) | 5979 | 6044 | + | 66 |  | TGC | 8 |
| trnR(tcg) | 6053 | 6121 | + | 69 |  | TCG | 26 |
| trnN(gtt) | 6148 | 6215 | + | 68 |  | GTT | 0 |
| TrnS1(tct) | 6216 | 6283 | + | 68 |  | TCT | 1 |
| trnE(ttc) | 6285 | 6351 | + | 67 |  | TTC | -2 |
| trnF(gaa) | 6350 | 6416 | - | 67 |  | GAA | -1 |
| nad5 | 6416 | 8122 | - | 1707 | TTG/TAA |  | 8 |
| trnH(gtg) | 8131 | 8198 | - | 68 |  | GTG | 0 |
| nad4 | 8199 | 9534 | - | 1336 | ATG/TGA |  | -7 |
| nad4l | 9528 | 9818 | - | 291 | ATG/TAA |  | 1 |
| trnT(tgt) | 9820 | 9884 | + | 65 |  | TGT | 0 |
| trnP(tgg) | 9885 | 9951 | - | 67 |  | TGG | 2 |
| nad6 | 9954 | 10457 | + | 504 | ATT/TAA |  | 3 |
| cob | 10461 | 11603 | + | 1143 | ATG/TAA |  | 3 |
| trnS2(tga) | 11607 | 11675 | + | 69 |  | TGA | 20 |
| nad1 | 11696 | 12646 | - | 951 | ATT/TAG |  | 0 |
| trnL1(tag) | 12647 | 12713 | - | 67 |  | TAG | -23 |
| rrnL | 12691 | 14073 | - | 1383 |  |  | -14 |
| trnV(tac) | 14060 | 14129 | - | 70 |  | TAC | 0 |
| rrnS | 14130 | 14953 | - | 824 |  |  | 0 |
| A+T rich region | 14954 | 15982 |  | 1029 |  |  |  |

E. RSCU of four dung beetles

|  | | *Catharsius molossus* | | *Liatongus bucerus* | | *Copris magicus* | | *Onitis falcatus* | |
| --- | --- | --- | --- | --- | --- | --- | --- | --- | --- |
| Amino acid | Codon | Number | RSCU | Number | RSCU | Number | RSCU | Number | RSCU |
| Phe | UUU(F) | 330 | 1.77 | 299 | 1.79 | 399 | 1.64 | 306 | 1.77 |
|  | UUC(F) | 43 | 0.23 | 35 | 0.21 | 87 | 0.36 | 39 | 0.23 |
| Leu | UUA(L) | 417 | 4.52 | 477 | 4.89 | 185 | 3.47 | 449 | 4.47 |
|  | UUG(L) | 37 | 0.4 | 23 | 0.24 | 50 | 0.94 | 39 | 0.39 |
|  | CUU(L) | 52 | 0.56 | 39 | 0.4 | 39 | 0.73 | 60 | 0.6 |
|  | CUC(L) | 6 | 0.06 | 2 | 0.02 | 9 | 0.17 | 6 | 0.06 |
|  | CUA(L) | 35 | 0.38 | 44 | 0.45 | 34 | 0.64 | 43 | 0.43 |
|  | CUG(L) | 7 | 0.08 | 0 | 0 | 3 | 0.06 | 6 | 0.06 |
| Ile | AUU(I) | 362 | 1.86 | 380 | 1.84 | 367 | 1.72 | 368 | 1.85 |
|  | AUC(I) | 28 | 0.14 | 34 | 0.16 | 60 | 0.28 | 29 | 0.15 |
| Met | AUA(M) | 215 | 1.76 | 252 | 1.83 | 136 | 1.49 | 236 | 1.76 |
|  | AUG(M) | 30 | 0.24 | 24 | 0.17 | 47 | 0.51 | 32 | 0.24 |
| Val | GUU(V) | 67 | 2.03 | 70 | 1.99 | 38 | 1.97 | 73 | 1.97 |
|  | GUC(V) | 6 | 0.18 | 4 | 0.11 | 7 | 0.36 | 4 | 0.11 |
|  | GUA(V) | 55 | 1.67 | 61 | 1.73 | 28 | 1.45 | 67 | 1.81 |
|  | GUG(V) | 4 | 0.12 | 6 | 0.17 | 4 | 0.21 | 4 | 0.11 |
| Ser | UCU(S) | 110 | 2.29 | 105 | 2.63 | 57 | 1.05 | 99 | 2.51 |
|  | UCC(S) | 13 | 0.27 | 11 | 0.28 | 43 | 0.79 | 22 | 0.56 |
|  | UCA(S) | 104 | 2.17 | 84 | 2.1 | 90 | 1.66 | 67 | 1.7 |
|  | UCG(S) | 3 | 0.06 | 4 | 0.1 | 18 | 0.33 | 5 | 0.13 |
|  | AGU(S) | 36 | 0.75 | 24 | 0.6 | 57 | 1.05 | 21 | 0.53 |
|  | AGC(S) | 15 | 0.31 | 9 | 0.23 | 44 | 0.81 | 1 | 0.03 |
|  | AGA(S) | 79 | 1.65 | 71 | 1.78 | 61 | 1.12 | 97 | 2.46 |
|  | AGG(S) | 24 | 0.5 | 12 | 0.3 | 65 | 1.2 | 3 | 0.08 |
| Pro | CCU(P) | 63 | 2.17 | 72 | 2.22 | 25 | 1.19 | 76 | 2.45 |
|  | CCC(P) | 26 | 0.9 | 16 | 0.49 | 25 | 1.19 | 18 | 0.58 |
|  | CCA(P) | 26 | 0.9 | 40 | 1.23 | 29 | 1.38 | 29 | 0.94 |
|  | CCG(P) | 1 | 0.03 | 2 | 0.06 | 5 | 0.24 | 1 | 0.03 |
| Thr | ACU(T) | 85 | 2.01 | 96 | 2.21 | 43 | 1.1 | 96 | 2.21 |
|  | ACC(T) | 19 | 0.45 | 15 | 0.34 | 37 | 0.94 | 15 | 0.34 |
|  | ACA(T) | 60 | 1.42 | 62 | 1.43 | 61 | 1.55 | 63 | 1.45 |
|  | ACG(T) | 5 | 0.12 | 1 | 0.02 | 16 | 0.41 | 0 | 0 |
| Ala | GCU(A) | 71 | 2.06 | 76 | 2.05 | 18 | 1.24 | 87 | 2.45 |
|  | GCC(A) | 17 | 0.49 | 19 | 0.51 | 8 | 0.55 | 15 | 0.42 |
|  | GCA(A) | 47 | 1.36 | 50 | 1.35 | 28 | 1.93 | 38 | 1.07 |
|  | GCG(A) | 3 | 0.09 | 3 | 0.08 | 4 | 0.28 | 2 | 0.06 |
| Tyr | UAU(Y) | 189 | 1.75 | 171 | 1.79 | 251 | 1.64 | 174 | 1.78 |
|  | UAC(Y) | 27 | 0.25 | 20 | 0.21 | 56 | 0.36 | 21 | 0.22 |
| His | CAU(H) | 47 | 1.77 | 62 | 1.72 | 30 | 1.71 | 62 | 1.75 |
|  | CAC(H) | 6 | 0.23 | 10 | 0.28 | 5 | 0.29 | 9 | 0.25 |
| Gln | CAA(Q) | 54 | 1.86 | 58 | 1.78 | 30 | 1.88 | 69 | 1.79 |
|  | CAG(Q) | 4 | 0.14 | 7 | 0.22 | 2 | 0.13 | 8 | 0.21 |
| Asn | AAU(N) | 201 | 1.68 | 189 | 1.77 | 283 | 1.6 | 187 | 1.86 |
|  | AAC(N) | 39 | 0.33 | 24 | 0.23 | 70 | 0.4 | 14 | 0.14 |
| Lys | AAA(K) | 91 | 1.49 | 104 | 1.86 | 125 | 1.46 | 102 | 1.7 |
|  | AAG(K) | 31 | 0.51 | 8 | 0.14 | 46 | 0.54 | 18 | 0.3 |
| Asp | GAU(D) | 59 | 1.79 | 55 | 1.69 | 34 | 1.51 | 53 | 1.66 |
|  | GAC(D) | 7 | 0.21 | 10 | 0.31 | 11 | 0.49 | 11 | 0.34 |
| Glu | GAA(E) | 53 | 1.61 | 67 | 1.81 | 25 | 1.79 | 65 | 1.83 |
|  | GAG(E) | 13 | 0.39 | 7 | 0.19 | 3 | 0.21 | 6 | 0.17 |
| Cys | UGU(C) | 44 | 1.73 | 38 | 1.85 | 53 | 1.14 | 31 | 1.82 |
|  | UGC(C) | 7 | 0.27 | 3 | 0.15 | 40 | 0.86 | 3 | 0.18 |
| Trp | UGA(W) | 73 | 1.66 | 81 | 1.8 | 74 | 1.19 | 79 | 1.84 |
|  | UGG(W) | 15 | 0.34 | 9 | 0.2 | 50 | 0.81 | 7 | 0.16 |
| Arg | CGU(R) | 16 | 1.25 | 12 | 0.87 | 3 | 0.55 | 20 | 1.67 |
|  | CGC(R) | 1 | 0.08 | 2 | 0.15 | 2 | 0.36 | 1 | 0.08 |
|  | CGA(R) | 29 | 2.27 | 35 | 2.55 | 15 | 2.73 | 21 | 1.75 |
|  | CGG(R) | 5 | 0.39 | 6 | 0.44 | 2 | 0.36 | 6 | 0.5 |
| Gly | GGU(G) | 56 | 1.33 | 32 | 0.65 | 16 | 0.56 | 44 | 0.9 |
|  | GGC(G) | 10 | 0.24 | 6 | 0.12 | 13 | 0.46 | 5 | 0.1 |
|  | GGA(G) | 74 | 1.76 | 103 | 2.09 | 54 | 1.89 | 96 | 1.97 |
|  | GGG(G) | 28 | 0.67 | 56 | 1.14 | 31 | 1.09 | 50 | 1.03 |
| ※ | UAA(*) | 27 | 1.54 | 12 | 1.85 | 124 | 1.5 | 26 | 1.49 |
|  | UAG(*) | 8 | 0.46 | 1 | 0.15 | 41 | 0.5 | 9 | 0.51 |
|  | codons | 3715 |  | 3710 |  | 3716 |  | 3713 |  |
